# Ultrasonic analysis of the anatomical relationships between vertebral arteries and internal jugular veins in children

**DOI:** 10.1111/j.1460-9592.2012.03816.x

**Published:** 2012-09

**Authors:** Kenji Kayashima, Masaya Ueki, Yuki Kinoshita, Brian Anderson

**Affiliations:** 1 Department of Anesthesia, Kyushukouseinenkin HospitalKitakyushu, Fukuoka, Japan; 2 Department of Anesthesia, Wakamatsu Hospital of the University of Occupational and Environmental HealthKitakyushu, Fukuoka, Japan; 3 Department of Anesthesia, Saiseikai Yahata General HospitalKitakyushu, Fukuoka, Japan

**Keywords:** child – Age, CVP lines – ultrasound, invasive – monitors – equipment, measurement, congenital heart disease – cardiac

## Abstract

**Background:**

Accidental puncture of the vertebral arteries (VAs) can occur through the internal jugular veins (IJVs) during central venous catheterization (CVC). We evaluated the anatomic relation of the VAs to the IJVs in children undergoing IJV cannulation.

**Methods:**

Fifty-five patients were placed in the supine position under general anesthesia. The right IJV, common carotid artery (CCA), and VA were described with an ultrasound probe perpendicular to all planes of the skin at the mid-portion between the suprasternal notch and mastoid process. The depth from the skin to VAs (D), width of the VAs (W), and distance from the IJVs to VAs (DIV) were measured. The extent of overlap between the IJVs and VAs was classified into overlapping, partially overlapping, and nonoverlapping. The risk was scored as 0–3 for each measurement. The scores were added and categorized into a low-risk group (L), 0–3, moderate-risk (M) group, 4–7; and high-risk (H) group, 8–10.

**Results:**

Mean (sd) age was 20.3 (33.9) months, height was 72.1 (26.0) cm, and weight was 8.9 (9.0) kg. The mean D, W, and DIV were 15.1 (3.3), 2.8 (1.1), and 4.6 (1.8) mm, respectively. Of the 55 patients, 7 were in group H, 33 in group M, and 15 in group L.

**Conclusions:**

Seven of the 55 children were categorized under the H group for accidental puncture of the VAs. Thus, it is important to identify the presence of the VAs to avoid accidental puncture during pediatric CVC.

## Introduction

Accidental puncture of the vertebral arteries (VAs) can occur during central venous catheterization (CVC) through the internal jugular veins (IJVs) (1–5). Deep insertion of the cannulation needle during catheterization of the IJV leads to accidental puncture of the VAs in adults (6). The common carotid arteries (CCAs) and IJVs in children were examined and distinguished using a small Doppler apparatus with a 2-mm diameter probe (7), before introducing an ultrasound apparatus, but it was difficult to detect VAs using the small Doppler apparatus. Little is known about the characteristics of VAs in children. In our experience, the VAs can be easily detected using an ultrasound device even in small children. Till date, no data about the anatomic relation between VAs and IJVs in children have been published. Therefore, in this study, we evaluate anatomic relation of the VAs with the IJVs in children undergoing IJV cannulation.

## Materials and methods

**Figure 1:**
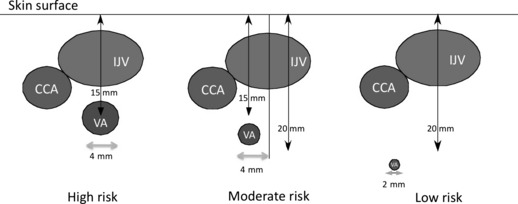
The relationships among the VA, CCA, and IJV position illustrated for high-, moderate-, and low-risk patients. VA, vertebral artery; CCA, common carotid artery; IJV, internal jugular vein.

**Figure 2:**
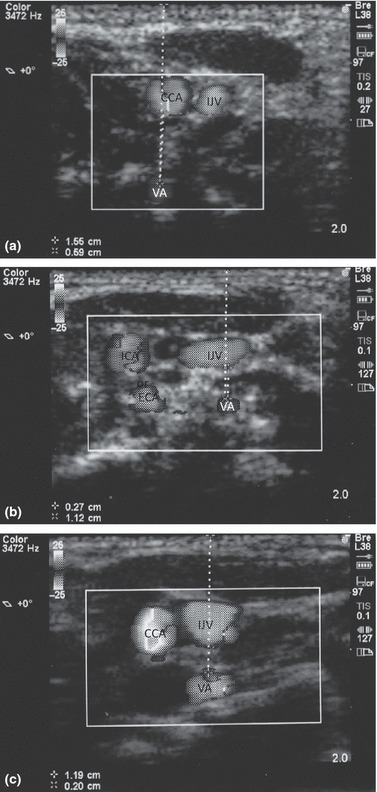
(a) An example image of a low-risk patient aged 1 month, 45 cm in height, and 3.1 kg in weight. (b) An example image of a moderate-risk patient aged 5 months, 53 cm in height, and 3.9 kg in weight. (c) An example image of a high-risk patient aged 3 months, 64 cm in height, and 5.4 kg in weight. VA, vertebral artery; CCA, common carotid artery; ECA, external carotid artery; ICA, internal carotid artery; IJV, internal jugular vein.

The ethics committee of our hospital approved this investigation, and written informed consent was obtained from the parents of all patients. Overall, 55 pediatric patients undergoing cardiac surgery under general endotracheal anesthesia from August 2008 to November 2009 were enrolled. The patients were placed in a supine position, and small rolled-up towels were put under their shoulders with their necks extended and rotated 15–30° to the left. The right VA, CCA, and IJV were described using an ultrasound apparatus containing a L10-5 MHz probe (TiTAN®; SonoSite co., Tokyo, Japan) placed perpendicular to all planes of the skin at the mid-portion between the suprasternal notch and mastoid process. The arteries were distinguished from the veins using the following indicators: direction of Doppler color flow and compression and palpation of the blood vessel. The VAs and IJVs were identified by two anesthesiologists, certified by the Japanese society of Anesthesiologists who have encountered more than 30 cases of adult CVC involving echo examination. The existence of the VAs was confirmed by their branching from the subclavian artery and disappearance near the fifth or sixth vertebral transverse process. The depth from the skin to the VAs (D), width of the VAs (W), and distance from the IJVs to VAs (DIV) were measured using the internal caliper of the ultrasound system on an optimized and frozen/still image. The extent of overlap between the VAs and IJVs were classified into overlapping, partially overlapping, and nonoverlapping (Figure 1). The risks were scored from 0 to 3 for each measurement, as shown in Table 1. The scores were added and used for the following categorization: low-risk (L) group from 0 to 3 (Figure 2a); moderate-risk (M) group, 4 to 7 (Figure 2b); and high-risk (H) group, 8 to10 (Figure 2c). All the IJV cannulations for the CVC were performed using real-time ultrasound guidance and an out-of-plane (short axis) technique. Complications such as accidental puncture of the CCAs or VAs, pneumothorax, and hemothorax were recorded.

**Table 1:** Risk scores for accidental puncture of the vertebral artery

Score	0	1	2	3
D	Over 20.1 mm	15.1–20.0 mm	Below 15.0 mm	
W	Below 2.0 mm	2.1–4.0 mm	Over 4.1 mm	
DIV	Over 6.1 mm	4.1–6.0 mm	2.1–4.0 mm	Below 2.0 mm
VA position	No overlapping	Partially overlapping		Overlapping

D, distance from the skin to VA; W, width of the VA; DIV, distance from the IJV to VA; VA position refers to the relationships between the IJV and VA; VA, vertebral artery; IJV, internal jugular vein.

### Statistical analysis

Statistical analysis was performed using Stat View® 4.0 for Windows (Abacus Concepts, Berkeley, CA, USA). Among the three groups, data were analyzed using one-way analysis of variance with post hoc test (Tukey’s multiple comparison test) for age, height, and weight and chi-square test for overlapping. *P*-values <0.05 was considered significant.

## Results

**Figure 3:**
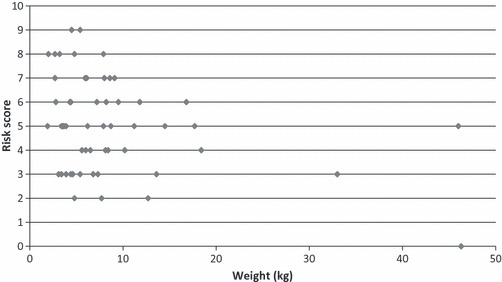
The relationships between patients’ weights and the risk scores of vertebral arterial puncture.

The mean (sd) age of 55 patients was 20.3 (33.9) months, height was 72.1 (26.0) cm, and weight was 8.9 (9.0) kg. The D, W, and DIV values were 15.1 (3.3), 2.8 (1.1), and 4.6 (1.8) mm, respectively. VAs were detected in 54 patients (98.2%); they could not be detected in a 120-month-old patient who was 142 cm tall and weighed 46.3 kg. Of the 55 patients, seven were in group H, 33 in group M, and 15 (including the one whom VAs were undetectable) in group L. Patients’ characteristics in each group are shown in Table 2. The VAs overlapped with the IJVs or partially overlapped in five patients (5/7, 71.4%) in group H, 14 (14/33, 42.4%) in group M, and 3 (3/15, 20.0%) in group L. No significant differences were noted in the results of the three groups with respect to age, height, weight, W, and extent of overlap. Significant differences between the D and DIV values of the L and M groups were noted (Table 2). The relationship between the weights and scores is described in Figure 3. All catheters were indwelled without complications such as punctures in the VAs or CCAs.

**Table 2:** Patients’ characteristics in each risk group

		Age	Height	Weight	D	W	DIV	Overlap
								
Group	*n*	Months	cm	kg	mm	mm	mm	*n*
H	7	4.4 ± 4.5 (4.0)	55.9 ± 12.5 (65.6)	4.4 ± 2.0 (6.0)	15.2 ± 2.3 (15.5)	3.3 ± 1.4 (2.8)	3.9 ± 1.3 (3.8)	5
M	33	20.2 ± 32.0 (11.5)	73.9 ± 24.5 (72.1)	8.8 ± 7.8 (8.2)	14.8 ± 3.6 (14.8)	2.9 ± 1.0 (2.9)	4.5 ± 1.8 (4.6)	14
L	15	28.6 ± 44.2 (5.0)	75.9 ± 32.3 (64.2)	11.2 ± 12.7 (5.6)	15.8 ± 3.1 (16.2)	2.3 ± 0.8 (2.4)	5.1 ± 2.2 (4.9)	3
*P* value		0.310	0.208	0.261	0.002	0.107	0.025	0.065
H vs L					*P* > 0.05		*P* > 0.05	
H vs M					*P* > 0.05		*P* > 0.05	
L vs M					*P* < 0.05		*P* < 0.05	

Data are the mean ± sd (median). No significant differences were noted in the results among the three groups in the patients' ages, heights, weights, W, and overlap. Significant differences between the D and DIV values of the L and M groups were noted. D, depth from the skin to VA; W, width of the VA; DIV, distance from the IJV to VA; H, high risk; M, moderate risk; L, low risk overlap: between the IJV and VA; VA, vertebral artery; IJV, internal jugular vein.

## Discussion

In this study, we examined the detailed positional relationships between the VAs and IJVs in pediatric CVC. Seven of the 55 cases (12.7%) were categorized under the H group for accidental puncture of the VAs. The IJVs were punctured without accidental puncture of any other arteries using the ultrasound apparatus in all 55 cases. While performing CVC in children, it is important to know the exact location of the VAs behind the IJVs.

Several cases of missing VAs have been reported in adults (8–13). Moseley and Sondheimer (13) refer to reports stating 1–9% absence of VAs on angiography. In contrast, VAs could not be detected in only one of our 55 cases (1.8%). Therefore, the presence of VAs should be checked carefully using Doppler color flow.

The origin of VAs is visible on ultrasonography in approximately 65–85% of adult patients (10). In the present study, it was quite straightforward to detect the origin of the VAs in children, because its position is usually shallow.

A 19.0-mm long 24-G puncture needle used in pediatric CVC advances 13.6 or 16.0 mm deep at maximum with a 45° or 60° insertion, respectively. In our study, the depth from the skin to anterior wall of the VAs averaged 15.1 mm and ranged from 9.4 to 26.9 mm; therefore, puncturing of the posterior wall of the IJVs could occur during pediatric CVC. The rate of puncturing the posterior wall of the IJV is 64% in adult mannequins (14). Because of its small diameter, a 24-G puncture needle causes little damage to the VAs even if punctured accidently; however, misplacement of a guidewire might lead to serious complications such as vertebral arteriovenous fistulas (1–4) or intra-aortic placement of a central venous catheter through a transverse cervical artery (15) in adults. In small infants, aspiration of blood often only occurs on needle withdrawal. Therefore, the vein is often deliberately penetrated (to aspirate blood), and this places the VAs at risk of puncture in a small group of infants. In cases in which the VAs are near the IJVs, using the ultrasonogram as a guide, the puncture needle should not be advanced toward the VAs (16).

The development of ultrasound apparatus has enabled us to easily detect small arteries such as the VAs. In the future, we will investigate the puncture rate of the posterior wall of the IJVs, as this type of puncture may be unavoidable in children.

Puncture of the VAs may occur more frequently than previously thought, and thus, the accepted practice of penetrating the posterior wall of the IJV may need to be re-evaluated.

The first limitation of this study is that it does not discuss the risk of accidental VA puncture. With the practice of real-time ultrasound guidance for CVC in children of all ages, the true risk of this rare complication is only speculative.

The second limitation concerns measurement: a minimum force was applied to avoid deforming the IJV shape when differentiating the arteries from the veins. Two anesthesiologists accustomed to the ultrasound apparatus obtained these data, and the measurement was successful.

The third limitation concerns the scoring system: the risk for accidental puncture of the VAs was categorized into three groups (H, M, and L groups) using the original scoring system, according to the examination of the images obtained using the ultrasound apparatus. After obtaining the data for D, W, and DIV, and the extent of overlap, we calculated the scores. We stratified the risk of accidental VA puncture using a scoring system that has not been validated.

In summary, seven of the 55 children (12.7%) were categorized under the H group of accidental puncture of the VAs. The IJV was punctured without accidental puncture of any other arteries using the ultrasound apparatus. It is important to detect the presence of the VAs to avoid accidental puncture during pediatric CVC.
